# Infection prevention and control in conflict-affected areas in northeast Syria: A cross-sectional study

**DOI:** 10.1016/j.ijregi.2024.100412

**Published:** 2024-07-29

**Authors:** Mohamed Alaa Ahmado, Muaaz Alghajar, Amina Olabi, Kristen Meagher, Preeti Patel, Abdulkarim Ekzayez

**Affiliations:** 1 Research for Health System Strengthening in Northern Syria (R4HSSS), Mehad, Erbil Mission, Iraq; 2 Mehad, The Headquarters, Paris, France; 3 Research for Health System Strengthening in Northern Syria (R4HSSS), King’s College London, London, UK; 4 Syria Public Health Network, London, UK

**Keywords:** Infection prevention and control (IPC), Infection Prevention and Control Assessment Framework (IPCAF), Hand Hygiene Self-Assessment Framework (HHSAF), Syria, Northeast Syria (NES), Conflict, Health assessment

## Abstract

**Objectives:**

In northeastern Syria (NES), the adherence of health care facilities to infection prevention and control (IPC) standards remains underexplored. This study evaluates the IPC performance of various health facilities against World Health Organization (WHO) benchmarks using the IPC Assessment Framework (IPCAF) and the Hand Hygiene Self-Assessment Framework (HHSAF).

**Methods:**

We conducted a cross-sectional survey of 33 health care facilities, including primary (PHC), secondary (SHC), and tertiary health care centres (THC). Data were collected via on-site evaluations using the IPCAF and HHSAF tools.

**Results:**

A significant 91% of facilities did not meet half of the WHO IPC minimum requirements. Specifically, 57% of PHCs met 26-50% of the standards, while none exceeded 75%. Among SHCs, 71% met 26-50% of the standards, while 44% of THCs fell within this range. Notably, 81.8% of facilities were classified as ‘inadequate’ per the IPCAF, with none achieving ‘intermediate’ or ‘advanced’ levels. The HHSAF results were similarly concerning, with 34.4% deemed ‘inadequate’ and 65.6% at the ‘basic’ level. A weak positive correlation (0.137) was found between IPCAF and HHSAF scores.

**Conclusions:**

NES health care facilities demonstrate substantial deficiencies in IPC compliance, with critical gaps in IPC programmes, health care–associated infection surveillance, and training. Urgent interventions are required to enhance IPC practices, leveraging local strengths and fostering international collaborations to improve patient safety and health care quality in the region.

## Highlights

• There is scarce research on infection prevention and control (IPC) in northeast Syria (NES).
• Health care in NES lacks basic IPC and hand hygiene practices.
• Most targeted health facilities in NES fail to meet half the minimum IPC standards.

## Introduction

In regions affected by conflict, the effects on the health care system can be far-reaching and devastating. This is particularly evident in northeastern Syria (NES), a home to an estimated 2.4 to 4 million people. The region has been profoundly affected by the Syrian conflict, the rise and eventual decline of the Islamic State of Iraq and Syria (ISIS), and continuing internal strife. Since 2011, NES has faced recurrent violence, internal displacement, and extensive damage to health facilities, which has exacerbated the public health crisis. The conflict has damaged health care infrastructure through attacks on health facilities and the repurposing of old buildings, limited access to medical services, a workforce exodus, weak governance, overcrowded refugee camps, inadequate sewage management, reliance on untreated water sources, and poor infection control practices. These factors have heightened the risk of infectious disease transmission [1,2]. Compared with the other areas in Syria, the humanitarian response in NES did not expand until the decline of ISIS in 2018. Even before the conflict, health care resource allocation in NES was marked by significant regional disparities, exemplified by the 2009 statistics indicating only less than one hospital bed per 1000 inhabitants in Raqqa, substantially fewer than the 2.6 beds per 1000 observed in Tartus and Latakia [3].

In this context, NES has seen a marked increase in infectious disease outbreaks, including water-borne diseases such as cholera [4]. The disruption of vaccination programmes and public health interventions, coupled with the damage to health care facilities, has led to the potential resurgence of vaccine-preventable diseases and the emergence of multidrug-resistant organisms [5]. Despite the conflict, NES offers a unique case study for public health research due to the presence of a centralised health governing body under the local administration, unlike some other parts of the country.

Historical outbreaks, such as the Ebola outbreak in West Africa before 2014, highlighted the role of health care settings in amplifying infections much more than community transmission, underscoring the need for effective infection prevention and control (IPC) programmes [6]. Effective IPC systems are fundamental to reducing health care–associated infections (HAIs), controlling antimicrobial resistance, and managing emerging pathogens, thus ensuring quality care as part of universal health coverage [7]. The effectiveness of IPC measures is intrinsically linked to the broader health system, encompassing service delivery, health workforce, health information systems, medical products and technologies, financing, and leadership and governance, as outlined by the World Health Organization (WHO) health system building blocks [8].

Evaluating IPC measures in conflict zones is crucial, providing insights that can inform the adaptation of successful strategies from other settings to address local weaknesses [9]. Given the spread of cholera and other infectious diseases in NES, a detailed examination of IPC provisions is essential for informing future outbreak responses. Despite anecdotal evidence of facility-level infection spread, no studies have documented this in NES or examined IPC practices in the region. This study aims to fill this gap, providing initial evidence on IPC in NES.

To address the unique health challenges faced by NES, this cross-sectional study examines the region's capacity to provide adequate IPC measures through a comprehensive assessment of critical health care facilities using the WHO's Infection Prevention and Control Assessment Framework (IPCAF) and Hand Hygiene Self-Assessment Framework (HHSAF) [10,11]. Additionally, the study investigates the correlation between IPCAF and HHSAF, offering insights into how overall IPC practices and specific hand hygiene practices interact and impact each other.

This work is part of the Research for Health System Strengthening in Syria (R4HSSS) programme, which aims to identify gaps and sustainable interventions, promote systems thinking, and adopt evidence-based approaches for the early recovery of the health system in northern Syria. The programme emphasises fostering equitable, multidisciplinary collaborations. Through collaboration with MEHAD, an organisation with local presence and reach to key actors, the research findings are rendered more applicable.

## Methodology

This study used a cross-sectional design to assess the IPC measures and hand hygiene practices implemented in selected health facilities across the four governorates in NES: Deir ez-Zor, Aleppo (specifically the areas under the control of the Syrian Democratic Forces, outside the Government of Syria's control), Raqqa, and Al-Hasakah. These regions are governed by the Autonomous Administration of North and East Syria (AANES), a semi-autonomous governing body established during the Syrian war [12].

NES is home to approximately 2.6 million people, with a diverse population that includes Kurds, Arabs, Assyrians, and other ethnic groups. In these areas, 2.6 out of 3.16 million people today are facing humanitarian needs, highlighting the severe impact of prolonged conflict and instability on the local population. Additionally, 662,000 people remain internally displaced, having fled from various parts of Syria because of ongoing violence and insecurity. Among the displaced, 293,000 continue to reside in last resort sites such as camps, informal settlements, and collective centres, which often lack adequate infrastructure and services [12].

### Selection of health facilities

The selection of health facilities was based on various criteria. Geographical distribution to represent the different regions of NES, multiple stakeholders, safety and security, secured funds, commitment and cooperation, and health facility type were all considered. Priority was given to health facilities located in areas with stability and safety during the implementation period and with sustainable funding. All public secondary facilities, as well as those that agreed to participate in the study, were selected.

### The sample

**Figure 1:**
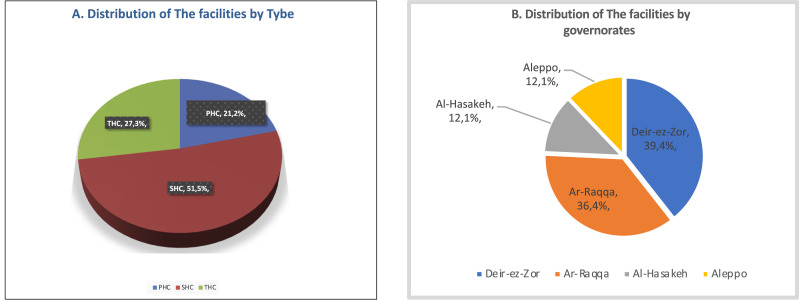
**A:** The selection sample contained 21.2%; (*n1*=7) primary health care centres (PHCs), 51.5%; (*n2*=17) secondary health care centres (SHCs), and 27.3%; (*n3*=9) tertiary health care centres (THCs) facilities. **B:** The geographical distribution, Deir-ez-Zor governorate had the highest proportion of the assessed facilities (39.4%; *n4*=13), followed by Ar-Raqqa (36.4%; *n5*=12), and lastly Al-Hasakeh and Aleppo (12.1%; *n6*=4 for each).

The selection of health care facilities in NES was designed to capture a spectrum of service levels and geographical locations. The NES forum definition was adopted to classify facilities into types. A detailed distribution is provided in Figures 1A and 1B. The selection of health facilities was supported and funded by four nongovernmental organisations (NGOs) and the local health authorities.

### Assessment tools

The assessment used two essential tools: the WHO IPCAF, as detailed in Appendix 1, and the WHO HHSAF, as detailed in Appendix 2. The IPCAF is a comprehensive questionnaire with a closed format and scoring system that can be used to measure and evaluate how well an IPC programme is being used in a facility [11]. Validating its efficacy, the effectiveness of the IPCAF tool has been rigorously tested, proving it to be a valuable instrument in the domain [13]. This tool has been extensively used in various studies encompassing a range of primary, secondary, and tertiary health care centres, affirming its applicability and relevance across different health care settings [14]. The key areas evaluated under IPCAF, also known as core components (CC), included the IPC programme (CC1), IPC guidelines (CC2), IPC education and training (CC3), HAI surveillance (CC4), multimodal strategies for implementation of IPC (CC5), monitoring/audits of IPC practices and feedback (CC6), workload, staffing, and bed occupancy (CC7), and built environment, materials, and equipment (CC8). Conversely, the HHSAF, composed of five components and 27 indicators, is designed to assess and improve hand hygiene practices in health care facilities [10]. The HHSAF framework has been tested for its ease of use and reliability, further bolstering its credibility in the field [15]. The key areas or components (C) evaluated under HHSAF included the system change (C1), training and education (C2), evaluation and feedback (C3), reminders in the workplace (C4), and an institutional safety climate (C5).

### Data collection

Data collection was systematically conducted by deploying three teams, each comprising a medical doctor and an assistant nurse, across the four governorates of NES. The teams underwent rigorous training, initially focusing on IPC principles and subsequently on the specific assessment tools used in this study. This training was designed to ensure their proficiency and consistency in applying the tools.

Collaboration with local organisations and health facility authorities was crucial. A memorandum of understanding (MoU) was established with the local authorities, and official cooperation requests were sent to the participating NGOs to facilitate smooth operations and enhance collaborative efforts. The health facility evaluations were conducted between April and May 2023.

The data collection methodology involved a multifaceted approach. Structured interviews were performed, adhering to a pre-defined question set to ensure uniformity and allow for systematic comparison across facilities. Observations were conducted to record real-time procedures and practices, providing a direct view of IPC implementation. Field visits were essential, offering an immersive examination of the physical environment, resource availability, and operational workflows within the facilities. Unlike other studies that relied on centralised reporting, our approach ensured that the data accurately reflected the actual conditions at each facility, minimising the risk of pre-visit adjustments that could distort the findings. This robust methodology underscores the study's commitment to capturing an authentic and comprehensive assessment of IPC practices in NES.

### Data analysis

Data analysis was performed using IBM SPSS Statistics, Version 29.0.10 and Microsoft Excel. Each core component of the IPCAF and the HHSAF was scored out of 100 points, yielding a maximum possible score of 800 for the IPCAF and 500 for the HHSAF. The grading system categorises overall compliance into four levels: inadequate, basic, intermediate, and advanced.

To assess the performance of each component, we calculated the median and interquartile range (IQR) to evaluate the distribution and variability of scores across different components. Component scores were classified using the following scale: inadequate (0-25), basic (25.1-50), intermediate (50.1-75), and advanced (75.1-100) [16]. This classification was consistently applied to both the IPCAF and HHSAF. Minimum IPC requirements were benchmarked against the WHO IPC standards and the 2019 global survey data [14,17]. The relationship between IPCAF and HHSAF scores was examined using Pearson correlation analysis, with a significance level set at *P* <0.05 and a 95% confidence interval (CI).

## Results

### Adherence to the WHO IPC minimum requirements

**Figure 2:**
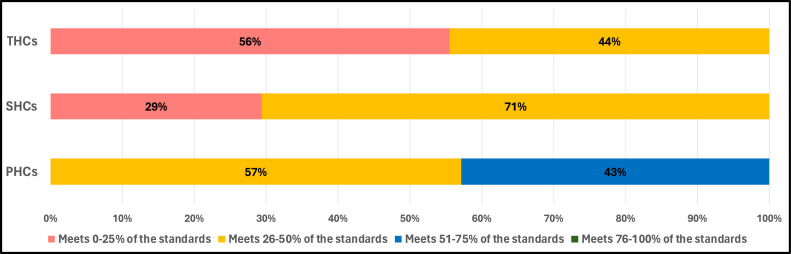
The percentage of health facilities that meet the World Health Organization (WHO) Infection Prevention and Control (IPC) minimum requirements according to the type of facility.

Of all the 33 surveyed facilities, 91% fell short of meeting half of the WHO IPC minimum requirements. Only three primary health care centres (PHCs) showed some promising results and met between 50% and 75% of minimum requirements, while 57% of PHCs met 26-50%. Among tertiary health care centres (THCs), only 56% met 0-25% of the standards and 44% met 26-50%. Secondary health care centres (SHCs) displayed a higher level of compliance, with 29% meeting 0-25% of the requirements and 71% meeting 26-50% (Figure 2).

### Classification of health facilities following IPCAF

In the IPCAF assessment, a high proportion (81.8%) of facilities fell into the ‘inadequate’ category. Only 18.2% of the facilities met the ‘basic’ standard, which is better than ‘inadequate’. None of the facilities attained the ‘intermediate’ or ‘advanced’ level. Approximately 34.4% of facilities’ hand hygiene practices were deemed ‘inadequate’. Most facilities (65.6%) achieved the ‘basic’ level.

### Implementation of the core components of the IPCAF

**Figure 3:**
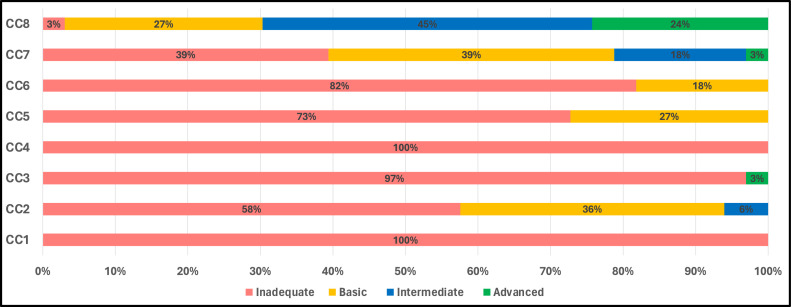
The percentage of health facilities classified (inadequate, basic, intermediate, and advanced) for each component of the Infection Prevention and Control Assessment Framework (IPCAF) tool.

The results provide a comprehensive breakdown of health care facilities’ alignment with the IPCAF components, offering a detailed spectrum from ‘inadequate’ to ‘advanced’. Components concerning IPC programmes (CC1) and HAI surveillance (CC4) stand out for their uniformity, with 100% of facilities classified as ‘inadequate’. Results for the IPC training and education component (CC3) were not much better, with 97% of facilities landing in the ‘inadequate’ category, although 3% achieved an ‘advanced’ classification. Conversely, built environment, materials, and equipment (CC8) emerged as the best-performing component. Only 3% of facilities were rated ‘inadequate’, and a notable 69% achieved either ‘intermediate’ or ‘advanced’ classifications (Figure 3).

Regarding the summary statistics, the mean and median scores provide further insight into the performance spectrum of these facilities across components. The highest mean value is observed for built environment, materials and equipment (CC8), at 63.0, aligning with the earlier observation that this was the best-performing component. The lowest mean scores are associated with IPC programmes (CC1) and HAI surveillance (CC4), which are close to zero, re-emphasising the concerning performance in these areas. Considering the IQR provides a measure of statistical spread, indicating variability within the facilities’ scores: built environment, materials, and equipment (CC8) again stands out, with a broader IQR of 50.0-75.5, signifying more significant variability in scores within this component. In addition, multimodal strategies for implementation of IPC (CC5), IPC education and training (CC3), and monitoring/audits of IPC practices and feedback (CC6) have low values at a median of 5.0, 10.0, and 15.0, respectively, emphasising uniform inadequacy across facilities. The median for all components is 167.5 (an inadequate level), reflecting the overall performance of the facilities when all aspects are considered (Table 1).

**Table 1:** Distribution of results of the total IPCAF score and scores per core component (mean, median, and interquartile range).

	CC1	CC2	CC3	CC4	CC5	CC6	CC7	CC8	Total
Mean	0.8	28.0	12.0	0.5.0	14.0	16.0	33.0	63.0	167.0
Median	0.0	25.5	10.0	0.0	5.0	15.0	30.0	65.0	167.5
Interquartile range	(0.0-0.0)	(15.0-40.0)	(0.0-15.0)	(0.0-0.0)	(0.0-30.0)	(10.0-25.0)	(15.0-47.5)	(50.0-75.5)	(127.5-190.0)

CC1, IPC program; CC2, IPC guidelines; CC3, IPC education and training; CC4, health care–associated infection surveillance; CC5, multimodal strategies for implementation of IPC; CC6, monitoring/audits of IPC practices and feedback; CC7, workload, staffing, and bed occupancy; CC8, built environment, materials, and equipment; IPC, infection prevention and control; IPCAF, IPC Assessment Framework.

### Implementation of the core components of the HHSAF

**Figure 4:**
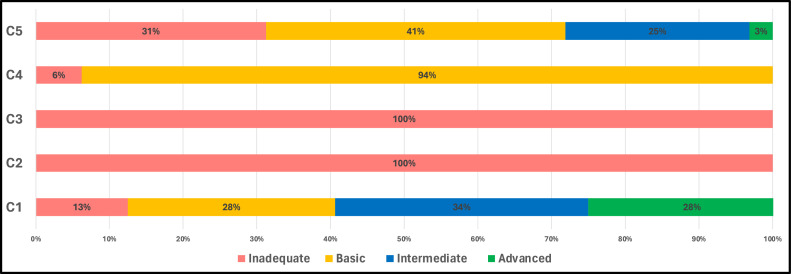
The percentage of health facilities classified (inadequate, basic, intermediate, and advanced) for each component of the Hand Hygiene Self-Assessment Framework (HHSAF).

A striking observation was the unanimous ‘inadequate’ classification for training and education (C2) and evaluation and feedback (C3), with both components rated at 100%. Conversely, the vast majority (94%) of health facilities were classified as ‘basic’. C1 or system change also displayed a more balanced distribution across classifications (Figure 4).

The mean for C2 or training and education is 6.9, which is particularly worrisome and reflective of the pressing inadequacies within this component. System change C1 and institutional safety climate C5, with respective means of 58.1 and 60.0, emerge as the components in which health facilities have invested the most effort. A cumulative median score of 157.5 (basic level) indicates the average performance of the surveyed facilities, with a relatively broad IQR of 87.5-191.2 (Table 2).

**Table 2:** Distribution of total HHSAF score results per core component (mean, median, and interquartile range).

	C1	C2	C3	C4	C5	Total
Mean	58.1	6.9	21.2	16.2	61.2	144.0
Median	60.0	5.0	20.0	20.0	45.0	157.5
Interquartile range	(40.0-77.5)	(0.0-7.5)	(15.0-30.0)	(0.0-25.0)	(25.0-56.2)	(87.5-191.2)

C1, system change; C2, training and education; C3, evaluation and feedback; C4, reminders in the workplace; C5, an institutional safety climate; HHSAF, Hand Hygiene Self-Assessment Framework.

### Correlation study results between the HHSAF and the IPCAF

In this study, we investigated the correlation between HHSAF and IPCAF scores using Pearson's correlation method. The results indicated a weak positive correlation of 0.137 between HHSAF and IPCAF scores, suggesting a slight increase in HHSAF values as IPCAF values increase, and vice versa. However, this correlation was not statistically significant at conventional levels (typically *P* <0.05) (Table 3).

**Table 3:** Correlation between IPCAF and HHSAF scores.

		IPCAF	HHSAF
IPCAF	Pearson correlation	1	0.137
	Significance (two-tailed)		0.446
	N	33	33
HHSAF	Pearson correlation	0.137	1
	Significance (two-tailed)	0.446	
	N	33	33

HHSAF, Hand Hygiene Self-Assessment Framework; IPCAF, Infection Prevention and Control Assessment Framework.

**Figure 5:**
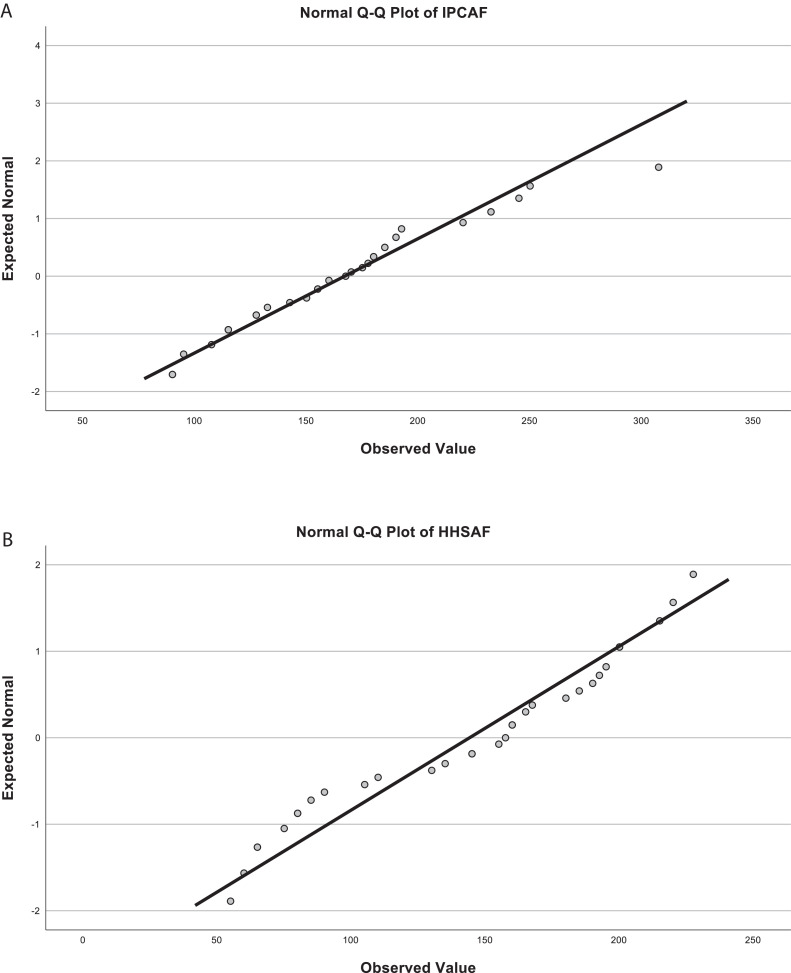
**A**. Normal Q-Q Plot of Infection Prevention and Control Assessment Framework (IPCAF) Scores. **B**. Normal Q-Q Plot of Hand Hygiene Self-Assessment Framework (HHSAF) Scores.

The decision to use Pearson's correlation was based on a thorough review of the literature and the nature of the variables involved. To assess the normality of the data, we performed both Kolmogorov–Smirnov and Shapiro–Wilk tests. The normality tests revealed a normal distribution for IPCAF (Kolmogorov–Smirnov: *P* = 0.200, Shapiro–Wilk: *P* = 0.240) and a non-normal distribution for HHSAF (Kolmogorov–Smirnov: *P* = 0.191, Shapiro–Wilk: *P* = 0.039). This was further confirmed by Q–Q plots, which showed that IPCAF data closely follow the normal distribution line, whereas HHSAF data show slight deviations from normality (Figures 5A and 5B). The correlation table is included for further reference (Table 4).

**Table 4:** Tests of normality for IPCAF and HHSAF scores.

	Kolmogorov–Smirnov^a^			Shapiro–Wilk		
	Statistic	df	Significance	Statistic	df	Significance
IPCAF	0.125	33	0.200^b^	0.959	33	0.240
HHSAF	0.127	33	0.191	0.932	33	0.039

HHSAF, Hand Hygiene Self-Assessment Framework; IPCAF, Infection Prevention and Control Assessment Framework.

a Lilliefors significance correction; ^b^ This is a lower bound of the true significance.

## Discussion

This study, concentrating on the adherence of health facilities to IPC standards in NES, offers in-depth perspective in an environment where similar studies are noticeably absent, and provides a contribution to the academic discourse in a context where analogous research is markedly scarce. In summary, our findings reveal the severe underperformance of the surveyed health care facilities in meeting the stipulated IPC minimum requirements. We found that 91% (equivalent to 30 of the 33 examined facilities) did not achieve half of the WHO's IPC minimum requirements. A detailed analysis reveals that PHCs show marginal adherence, with 57% meeting only 0-50% of the benchmarks. Conversely, 43% (representing three distinct facilities) adhered to 51-75% of the standards. Furthermore, both SHCs and THCs exhibited deficiencies. Every SHC and THC assessed (100% in both categories) failed to meet more than half of the IPC standards.

To our knowledge, the interconnection of the tools used in this study has only been documented in one other instance [14]. It is essential to highlight that, within the broader Syrian context, this represents the first effort, as there is a paucity of published studies that systematically assess IPC practices across the region's health care landscape. Furthermore, the importance of our findings is heightened given that most health facilities under investigation by IPCAF and HHSAF were classified as ‘inadequate’ or ‘basic’. The classification of health facilities as ‘inadequate’ or ‘basic’ is of concern. ‘Inadequate’ suggests that the facilities might need to improve in meeting even the most fundamental IPC standards, posing considerable risks to patients and health care professionals. Meanwhile, ‘basic’ implies that while some foundational measures might be in place, they need to be more comprehensive to meet the comprehensive standards required to ensure optimal patient safety and service quality. Such classifications indicate a potential for increased infections, compromised patient care, and reduced trust in the health care system.

Our study differed methodologically from other studies published on the topic, lending robustness to the findings in various ways. Firstly, the selection of facilities was predicated on well-defined criteria, eschewing the self-selecting surveys commonly observed in broader global assessments; this approach could skew the global IPCAF and HHSAF scores towards an overestimation [14,[18], [19], [20]]. It is worth noting that extant literature, particularly studies contextualised to specific regions, has also used specialised criteria for facility selection. Secondly, evaluations were undertaken through on-site visits by our dedicated teams, who received comprehensive IPC training focusing on the tools under study. This represents a new approach relative to the dominant methodology in the literature, which frequently relies on self-survey mechanisms [14,[18], [19], [20]]. We assume this approach is more appropriate for health environments in conflict areas, where many facilities lack IPC specialists [21]. This stance aligns seamlessly with our findings.

The inadequacy starkly contrasts with the global trend, as illuminated by the global survey data from 2019 [14]. This comprehensive survey, encompassing 4440 health care facilities in 81 countries across all six WHO regions and spanning diverse income levels, showcased that 92.9% of health facilities worldwide met at least half of the assessed indicators. This further underscores the pressing need for enhanced efforts and targeted strategies to bridge this significant NES health care quality gap. The worldwide data reveal that 15.2% of all health facilities met 100% of the minimum requirements. While this number may seem modest, it becomes even more significant when considering that this figure plunges to 0% in low-income countries [14].

Reflecting on our results by classifying the surveyed health facilities according to the tools used, the vast majority (81.8%) of the surveyed facilities failed to meet the basic criteria for IPC, resulting in their classification as ‘inadequate’. While 18.2% managed to achieve a ‘basic’ level, none could reach the ‘intermediate’ or ‘advanced’ thresholds. A more detailed analysis of the IPC components reveals that the lowest scores were attributed to the first (IPC programme) and fourth (HAI surveillance) components, followed by the fifth (multimodal strategies for implementing IPC) and third (IPC training and education) components. These results highlight potential areas that require urgent attention and resource allocation. Conversely, the highest scores were in the eighth (built environment, materials, and equipment) and the seventh (workload, staffing, and bed occupancy) components. This implies that while structural and material infrastructure is in better condition, IPC programmes, HAI surveillance, training, and improved multimodal strategy mechanisms require immediate enhancement. It corroborates a 2018 study that emphasised the establishment of a robust surveillance HAI system in Syria [5]. This pronounced inadequacy is especially concerning when juxtaposed with global standards. Globally, the 2019 survey indicates a clear correlation between a nation's economic status and IPC standards, with high-income countries often achieving the ‘advanced’ IPC level. In contrast, low-income countries hovered around a ‘basic’ level with a median of 385.0, while our results showed a median of 167.5 (an inadequate level). However, this economic gradient in IPC standards does not uniformly dictate the quality of IPC practices, as variations are observed even within similar income brackets [14]. While countries such as Germany and Austria showcased IPC results consistent with their high-income status, which is not comparable to the situation in NES, countries with contrasting economic capabilities portrayed mixed results [22,23]. For instance, Ghana, despite its partial completion of the IPCAF assessment, reported a majority of facilities at ‘basic’ or ‘intermediate’ levels [24]. In stark contrast, hospitals in Islamabad, Pakistan mirrored NES's dire situation, being labelled ‘inadequate’ [25]. However, despite their economic challenges, nations such as India and Türkiye reported many facilities reaching ‘advanced’ or ‘intermediate’ IPC levels, suggesting that economic constraints are not the sole determinants of effective IPC practices [26]. However, the situation in NES cannot be compared to either countries given the difference in health system capacity. The scenario in Afghanistan and Côte d'Ivoire further solidifies this observation, where, despite economic limitations, facilities varied widely in their IPC implementation, ranging from ‘inadequate’ to ‘intermediate’ [27,28]. An analysis of the global results concerning specific IPC components further accentuates the standard divergence. The eighth component (built environment, materials, and equipment for IPC) globally scored the highest, a trend also observed in NES. Core component 3 (IPC education and training) was among the lowest-scoring globally and in NES, emphasising the pronounced need for bolstered IPC education in the region [14]. The vast chasm in scores for core components 1 and 4 between low-income countries and their high-income counterparts further amplifies the challenges that regions such as NES face in this domain. In light of these findings, it is believed that understanding the gaps in IPC, and subsequently addressing them, could have implications for the wider health system in NES, by offering a cost-effective public health solution to issues that, if left unaddressed, can place great burden on a health system that is already overextended because of years of conflict.

In light of the present study, we elucidated the state of hand hygiene standards in NES by leveraging HHSAF. Our observations reveal a concerning picture. Out of the 33 health facilities surveyed, 34.4% were found to be ‘inadequate’ regarding HHSAF levels, while most (65.6%) were at the ‘basic’ level, with a median score of 157.5. All facilities have yet to reach the ‘intermediate’ or ‘advanced’ tiers, emphasising the region's stark need for comprehensive hand hygiene interventions. When juxtaposed with global trends, the results from NES lag markedly. The global survey from 2015 identified hand hygiene levels at 229 (basic level) for low-income countries, which saw a slight uptick in 2019 to 233. Even more notably, high-income countries demonstrated hand hygiene levels at 381 (advanced level) in 2015, elevating to 395 in 2019. These figures accentuate the disparities between NES and low- and high-income countries globally [20]. The HHSAF component assessment for NES presented critical insights. While ‘system change’ and ‘institutional safety climate for hand hygiene’ secured relatively high medians of 60.0 and 45, respectively, the domain ‘training and education’ drastically underperformed, with a median of 5.0 points. This underscores the dire need for enhanced hand hygiene education initiatives in the region. Moreover, in contrast with the 2019 global survey, the ‘institutional safety climate’ was universally identified as the lowest-scoring component. Yet, in the case of Syria, it was one of the stronger components, suggesting unique regional challenges in other domains, especially in training and education [21]. The comparative scenario in other countries further highlights NES's unique predicament. For instance, Cambodian hospitals in 2020 were predominantly at the ‘basic’ level, mirroring NES's prevalent trend, despite the marked differences in settings [29]. However, Sierra Leone, a setting that is also regularly faced with disease outbreaks, showcased progress in 2021, with most hospitals achieving an ‘intermediate’ level, indicating possible pathways for improvement [30]. Adapting these findings into programmes to improve hand hygiene levels in NES is of high relevance, especially given the frequent disease outbreaks in the area. Therefore, strengthening hand hygiene practices and infrastructure would be of impact in the long term and would play a pivotal role in health system preparedness.

Regarding our results of the association between HHSAF and IPCAF, our study revealed a weak positive correlation between HHSAF and IPCAF scores, diverging from global trends where a strong correlation exists between IPC implementation and hand hygiene [14]. We assume the explanation for our distinct findings is that the health facilities we surveyed largely lacked the foundational IPC programme component (CC1), making them atypical and possibly less reflective of the global trend in this correlation.

In light of our findings, a comprehensive strategy for IPC in the NES is needed to prioritise the adoption of WHO IPC standards. Such a strategy should focus on ensuring long-term improvements in patient and health care worker safety.

Such a strategy cannot be implemented without greater investment in IPC education and training. There is a significant deficit in IPC training within NES, necessitating targeted funding for context-specific training programmes. Insights from successful IPC models in similar low-resource settings should inform these initiatives.

Enhancing HAI surveillance systems is critical. Developing robust mechanisms for monitoring and reporting HAIs will facilitate timely interventions and help mitigate infection risks. Similarly, addressing hand hygiene practices is a priority. Given the alarming scores in hand hygiene assessments, comprehensive training programmes must be introduced to bring NES facilities in line with global standards.

It is also important to leverage local strengths. Despite notable gaps, NES has strengths in areas such as infrastructure and the institutional safety climate for hand hygiene. These strengths should be leveraged to set benchmarks and guide improvements across other sectors.

Further research into the specific challenges faced in NES is necessary. Additional studies should be funded to provide insights that inform tailored interventions and enhance evidence-based practices.

Finally, fostering collaborations with countries that have made progress in IPC despite similar socio-economic challenges can be valuable. Collaborative ventures should promote knowledge exchange and the adoption of best practices.

## Conclusion

This study highlights that most facilities, especially SHCs and THCs, must at least catch up to meet WHO's minimum IPC requirements. Comparatively, primary health care centres present a slightly more optimistic scenario, albeit still underperforming relative to global trends. In hand hygiene, our results further underline NES's challenges, with most facilities falling under ‘inadequate’ or ‘basic’ IPC levels. Our findings emphasise the urgent need for improvement of IPC programmes, HAI surveillance, multimodal strategies, and training in NES, necessitating immediate attention and resource allocation. Moreover, the distinctive challenges posed in the domain of ‘training and education’ for hand hygiene in Syria, relative to global trends, underscore region-specific impediments that require tailored interventions. NES's health care facilities need more IPC measures and hand hygiene standards. Through this study, we attempt to lay the foundation for future work. This study acts as a ‘phase one’ for next steps aiming at bridging these gaps, which is critical for the region's patient safety and health service quality and for establishing a robust health care infrastructure that can withstand current and future challenges. The upcoming steps will take place in the form of designing IPC programmes to implement as interventions within an operational research framework, where the process is documented and lessons learned disseminated with key stakeholders in NES. The pressing need for more research in the Syrian context is evident, as the data reveal areas for improvement and offer direction for future interventions and policies.

### Limitations

The generalisability of this assessment may be limited to the selected health facilities in NES. Thus, the results may not fully represent the wider region. Whilst most secondary-level public facilities were represented, facilities that did not agree to participate were excluded. This may have introduced bias, leading to limited generalisability.

Furthermore, the facilities were not repeatedly visited, which could reduce the reliability of the data, as the tests were only taken once. Longer study periods or repeated visits could have contributed to reducing this bias. This was not avoidable given the resource restrictions of the study. Bias could also have been introduced by many different researchers being involved in the data collection.

The validity of the data could have also been affected by external factors such as resources available to the facility at the given time. Conclusions and comparisons between different facilities were kept conservative to mitigate this risk of low internal validity of the data.

## Declarations of competing interest

The authors have no competing interests to declare.

## Appendix Supplementary materials

Image, application 1

Image, application 2
