# Supplementary material for: Infection prevention and control in conflict-affected areas in northeast Syria: A cross-sectional study
Source: IJID Reg. 2024 Jul 29;12:100412. doi: 10.1016/j.ijregi.2024.100412 (PMC11415633; doi:10.1016/j.ijregi.2024.100412)
Supplement: Supplementary file 2 [file mmc2.pdf]

World Health Organization

SAVE LIVES
Clean **Your** Hands

Hand Hygiene Self-Assessment Framework 2010

Introduction and user instructions

The Hand Hygiene Self-Assessment Framework is a systematic tool with which to obtain a situation analysis of hand hygiene promotion and practices within an individual health-care facility.

What is its purpose?

While providing an opportunity to reflect on existing resources and achievements, the Hand Hygiene Self-Assessment Framework also helps to focus on future plans and challenges. In particular, it acts as a diagnostic tool, identifying key issues requiring attention and improvement. The results can be used to facilitate development of an action plan for the facility's hand hygiene promotion programme. Repeated use of the Hand Hygiene Self-Assessment Framework will also allow documentation of progress with time.

Overall, this tool should be a catalyst for implementing and sustaining a comprehensive hand hygiene programme within a health-care facility.

Who should use the Hand Hygiene Self-Assessment Framework?

This tool should be used by professionals in charge of implementing a strategy to improve hand hygiene within a health-care facility. If no strategy is being implemented yet, then it can also be used by professionals in charge of infection control or senior managers at the facility directorate. The framework can be used globally, by health-care facilities at any level of progress as far as hand hygiene promotion is concerned.

How is it structured?

The Hand Hygiene Self-Assessment Framework is divided into five components and 27 indicators. The five components reflect the five elements of the WHO Multimodal Hand Hygiene Improvement Strategy (http://www.who.int/gpsc/5may/tools/en/index.html) and the indicators have been selected to represent the key elements of each component. These indicators are based on evidence and expert consensus and have been framed as questions with defined answers (either "Yes/No" or multiple options) to facilitate self-assessment. Based on the score achieved for the five components, the facility is assigned to one of four levels of hand hygiene promotion and practice: Inadequate, Basic, Intermediate and Advanced.

Inadequate: hand hygiene practices and hand hygiene promotion are deficient. Significant improvement is required.

Basic: some measures are in place, but not to a satisfactory standard. Further improvement is required.

Intermediate: an appropriate hand hygiene promotion strategy is in place and hand hygiene practices have improved. It is now crucial to develop long-term plans to ensure that improvement is sustained and progresses.

Advanced: hand hygiene promotion and optimal hand hygiene practices have been sustained and/or improved, helping to embed a culture of safety in the health-care setting.

Leadership criteria have also been identified to recognise facilities that are considered a reference centre and contribute to the promotion of hand hygiene through research, innovation and information sharing. The assessment according to leadership criteria should only be undertaken by facilities having reached the Advanced level.

How does it work?

While completing each component of the Hand Hygiene Self-Assessment Framework, you should circle or highlight the answer appropriate to your facility for each question. Each answer is associated with a score. After completing a component, add up the scores for the answers you have selected to give a subtotal for that component. During the interpretation process these subtotals are then added up to calculate the overall score to identify the hand hygiene level to which your health-care facility is assigned.

The assessment should not take more than 30 minutes, provided that the information is easily available.

Within the Framework you will find a column called "WHO implementation tools" listing the tools made available from the WHO First Global Patient Safety Challenge to facilitate the implementation of the WHO Multimodal Hand Hygiene Improvement Strategy (http://www.who.int/gpsc/5may/tools/en/index.html). These tools are listed in relation to the relevant indicators included in the Framework and may be useful when developing an action plan to address areas identified as needing improvement.

Is the Hand Hygiene Self-Assessment Framework suitable for inter-facility comparison?

Health-care facilities or national bodies may consider adopting this tool for external comparison or benchmarking. However, this was not a primary aim during the development of this tool. In particular, we would draw attention to the risks inherent in using a self-reported evaluation tool for external benchmarking and also advise the use of caution if comparing facilities of different sizes and complexity, in different socioeconomic settings. It would be essential to consider these limitations if inter-facility comparison is to be undertaken.

All reasonable precautions have been taken by the World Health Organization to verify the information contained in this document. However, the published material is being distributed without warranty of any kind, either expressed or implied. The responsibility for the interpretation and use of the material lies with the reader. In no event shall the World Health Organization be liable for damages arising from its use.
WHO acknowledges the Hôpitaux Universitaires de Genève (HUG), in particular the members of the Infection Control Programme, for their active participation in developing this material.

World Health Organization

SAVE LIVES
Clean **Your** Hands

Hand Hygiene Self-Assessment Framework 2010

1. System Change

Question	Answer	Score	WHO improvement tools
**1.1** How easily available is alcohol-based handrub in your health-care facility? **Choose one answer**	Not available	0	→ Ward Infrastructure Survey → Protocol for Evaluation of Tolerability and Acceptability of Alcohol-based Handrub in Use or Planned to be Introduced:Method 1 → Guide to Implementation II.1
	Available, but efficacy[1] and tolerability[2] have not been proven	0	
	Available only in some wards or in discontinuous supply (with efficacy[1] and tolerability[2] proven)	5	
	Available facility-wide with continuous supply (with efficacy[1] and tolerability[2] proven)	10	
	Available facility-wide with continuous supply, and at the point of care[3] in the majority of wards (with efficacy[1] and tolerability[2] proven)	30	
	Available facility-wide with continuous supply at each point of care[3] (with efficacy[1] and tolerability[2] proven)	50	
**1.2** What is the sink:bed ratio? **Choose one answer**	Less than 1:10	0	→ Ward Infrastructure Survey → Guide to Implementation II.1
	At least 1:10 in most wards	5	
	At least 1:10 facility-wide and 1:1 in isolation rooms and in intensive care units	10	
**1.3** Is there a continuous supply of clean, running water[4]?	No	0	→ Ward Infrastructure Survey → Guide to Implementation II.1
	Yes	10	
**1.4** Is soap[5] available at each sink?	No	0	→ Ward Infrastructure Survey → Guide to Implementation II.1
	Yes	10	
**1.5** Are single-use towels available at each sink?	No	0	→ Ward Infrastructure Survey → Guide to Implementation II.1
	Yes	10	
**1.6** Is there dedicated/available budget for the continuous procurement of hand hygiene products (e.g. alcohol-based handrubs)?	No	0	→ Guide to Implementation II.1
	Yes	10	

Extra Question: Action plan

**Answer this question ONLY if you scored less than 100 for questions 1.1 to 1.6:** Is there realistic plan in place to improve the infrastructure[6] in your health-care facility?	No	0	→ Alcohol-based Handrub Planning and Costing Tool → Guide to Local Production: WHO-recommended Handrub Formulations → Guide to Implementation II.1
	Yes	5	
	**System Change subtotal**	**/100**	

1. Efficacy: The alcohol-based handrub product used should meet recognised standards of antimicrobial efficacy for hand antisepsis (ASTM or EN standards). Alcohol-based handrubs with optimal antimicrobial efficacy usually contain 75 to 85% ethanol, isopropanol, or n-propanol, or a combination of these products. The WHO-recommended formulations contain either 75% v/v isopropanol, or 80% v/v ethanol.

2. Skin tolerability: The alcohol-based handrub product is well tolerated by health-care workers skin (i.e. it does not harm or irritate the skin) when used in clinical care, as demonstrated by reliable data. The WHO Protocol for Evaluation of Tolerability and Acceptability of Alcohol-based Handrub in Use or Planned to be Introduced can be used as a reference.

3. Point of care: The place where three elements come together: the patient, the health-care worker, and care or treatment involving contact with the patient or his/her surroundings (within the patient zone). Point-of-care products should be accessible without having to leave the patient zone (ideally within arms reach of the health-care worker or within 2 meters).

4. Clean, running water: A water supply that is either piped in (or where this is not available, from onsite storage with appropriate disinfection) that meets appropriate safety standards for microbial and chemical contamination. Further details can be found in Essential environmental health standards in health care (Geneva, World Health Organization, 2008, http://whqlibdoc.who.int/publications/2008/9789241547239_eng.pdf).

5. Soap: Detergent-based products that contain no added antimicrobial agents, or may contain these solely as preservatives. They are available in various forms including bar soap, tissue, leaf, and liquid preparations.

6. Infrastructure: The “infrastructure” here referred to includes facilities, equipment, and products that are required to achieve optimal hand hygiene practices within the facility. Specifically, it refers to the indicators included in questions 1.1-1.5 and detailed in the WHO Guidelines on Hand Hygiene in Health Care 2009, Part I, Chapter 23.5 (e.g. availability of alcohol based handrub at all points of care, a continuous supply of clean, running water and a sink:bed ratio of at least 1:10, with soap and single-use towels at each sink).

All reasonable precautions have been taken by the World Health Organization to verify the information contained in this document. However, the published material is being distributed without warranty of any kind, either expressed or implied. The responsibility for the interpretation and use of the material lies with the reader. In no event shall the World Health Organization be liable for damages arising from its use.
WHO acknowledges the Hôpitaux Universitaires de Genève (HUG), in particular the members of the Infection Control Programme, for their active participation in developing this material.

Hand Hygiene Self-Assessment Framework 2010

2. Training and Education

Question	Answer	Score	WHO improvement tools
**2.1** Regarding training of health-care workers in your facility:			
2.1a How frequently do health-care workers receive training regarding hand hygiene[7] in your facility? **Choose one answer**	Never	0	→ Slides for Education Session for Trainers, Observers and Health-care Workers<br>→ Hand Hygiene Training Films<br>→ Slides Accompanying the Training Films<br>→ Slides for the Hand Hygiene Co-ordinator<br>→ Hand Hygiene Technical Reference Manual<br>→ Hand Hygiene Why, How and When Brochure<br>→ Guide to Implementation II.2
	At least once	5	
	Regular training for medical and nursing staff, or all professional categories (at least annually)	10	
	Mandatory training for all professional categories at commencement of employment, then ongoing regular training (at least annually)	20	
2.1b Is a process in place to confirm that all health-care workers complete this training?	No	0	
	Yes	20	
**2.2** Are the following WHO documents (available at www.who.int/gpsc/5may/tools), or similar local adaptations, easily available to all health-care workers?			→ Guide to Implementation II.2
2.2a The 'WHO Guidelines on Hand Hygiene in Health-care: A Summary'	No	0	→ WHO Guidelines on Hand Hygiene in Health Care: A Summary
	Yes	5	
2.2b The WHO 'Hand Hygiene Technical Reference Manual'	No	0	→ Hand Hygiene Technical Reference Manual
	Yes	5	
2.2c The WHO 'Hand Hygiene: Why, How and When' Brochure	No	0	→ Hand Hygiene Why, How and When Brochure
	Yes	5	
2.2d The WHO 'Glove Use Information' Leaflet	No	0	→ Glove Use Information Leaflet
	Yes	5	
**2.3** Is a professional with adequate skills[8] to serve as trainer for hand hygiene educational programmes active within the health-care facility?	No	0	→ WHO Guidelines on Hand Hygiene in Health Care<br>→ Hand Hygiene Technical Reference Manual<br>→ Hand Hygiene Training Films<br>→Slides Accompanying the Training Films<br>→ Guide to Implementation II.2
	Yes	15	
**2.4** Is a system in place for training and validation of hand hygiene compliance observers?	No	0	
	Yes	15	
**2.5** Is there is a dedicated budget that allows for hand hygiene training?	No	0	→ Template Letter to Advocate Hand Hygiene to Managers<br>→ Template Letter to communicate Hand Hygiene Initiatives to Managers<br>→ Template Action Plan<br>→ Guide to Implementation II.2 and III.1 (page 33)
	Yes	10	
	**Training and Education subtotal**	**/100**	

7. Training in hand hygiene: This training can be done using different methods but the information conveyed should be based on the WHO multimodal hand hygiene improvement strategy or similar material. Training should include the following:
- The definition, impact and burden of health care-associated infection (HCAI)
- Major patterns of transmission of health care-associated pathogens
- Prevention of HCAI and the critical role of hand hygiene
- Indications for hand hygiene (based on the WHO 'My 5 Moments for Hand Hygiene' approach)
- Correct technique for hand hygiene (refer to 'How to Handrub' and 'How to Hand Wash')

8. A professional with adequate skills: Medical staff or nursing staff trained in Infection Control or Infectious Diseases, whose tasks formally include dedicated time for staff training. In some settings, this could also be medical or nursing staff involved in clinical work, with dedicated time to acquire thorough knowledge of the evidence for and correct practice of hand hygiene (the minimum required knowledge can be found in the WHO Guidelines on Hand Hygiene in Health Care and the Hand Hygiene Technical Reference Manual).

All reasonable precautions have been taken by the World Health Organization to verify the information contained in this document. However, the published material is being distributed without warranty of any kind, either expressed or implied. The responsibility for the interpretation and use of the material lies with the reader. In no event shall the World Health Organization be liable for damages arising from its use.
WHO acknowledges the Hôpitaux Universitaires de Genève (HUG), in particular the members of the Infection Control Programme, for their active participation in developing this material.

3. Evaluation and Feedback

Question	Answer	Score	WHO improvement tools
**3.1** Are regular (at least annual) ward-based audits undertaken to assess the availability of handrub, soap, single use towels and other hand hygiene resources?	No	0	→ Ward Infrastructure Survey<br>→ Guide to Implementation II.3
	Yes	10	
**3.2** Is health care worker knowledge of the following topics assessed at least annually (e.g. after education sessions)?			
3.2a. The indications for hand hygiene	No	0	→ Hand Hygiene Knowledge Questionnaire for Health-Care Workers<br>→ Guide to Implementation II.3
	Yes	5	
3.2b. The correct technique for hand hygiene	No	0	
	Yes	5	
**3.3 Indirect Monitoring of Hand Hygiene Compliance**			
3.3a Is consumption of alcohol-based handrub monitored regularly (at least every 3 months)?	No	0	→ Soap/Handrub Consumption Survey<br>→ Guide to Implementation II.3
	Yes	5	
3.3b Is consumption of soap monitored regularly (at least every 3 months)?	No	0	
	Yes	5	
3.3c Is alcohol based handrub consumption at least 20L per 1000 patient-days?	No (or not measured)	0	
	Yes	5	
**3.4 Direct Monitoring of Hand Hygiene Compliance** Only complete section 3.4 if hand hygiene compliance observers in your facility have been trained and validated and utilise the WHO 'My 5 Moments for Hand Hygiene' (or similar) methodology			
3.4a How frequently is direct observation of hand hygiene compliance performed using the WHO Hand Hygiene Observation tool (or similar technique)?<br>**Choose one answer**	Never	0	→ WHO Hand Hygiene Observation form<br>→ Hand Hygiene Technical Reference Manual<br>→ Guide to Implementation II.3
	Irregularly	5	
	Annually	10	
	Every 3 months or more often	15	
3.4b What is the overall hand hygiene compliance rate according to the WHO Hand Hygiene Observation tool (or similar technique) in your facility?<br>**Choose one answer**	≤ 30%	0	→ Guide to Implementation II.3<br>→ Observation form<br>→ Data Entry Analysis tools<br>→ Instructions for Data Entry and Analysis<br>→ Epi Info™ software[9]<br>→ Data Summary Report Framework
	31 – 40%	5	
	41 – 50%	10	
	51 – 60%	15	
	61 – 70%	20	
	71 – 80%	25	
	≥ 81%	30	
**3.5 Feedback**			
3.5a **Immediate feedback** Is immediate feedback given to health-care workers at the end of each hand hygiene compliance observation session?	No	0	→ Guide to Implementation II.3<br>→ Observation and Basic Compliance Calculation forms
	Yes	5	
3.5b **Systematic feedback** Is regular (at least 6 monthly) feedback of data related to hand hygiene indicators with demonstration of trends over time given to:			→ Data Summary Report Framework<br>→ Guide to Implementation II.3
3.5b.i Health-care workers?	No	0	
	Yes	7.5	
3.5b.ii Facility leadership?	No	0	
	Yes	7.5	
	**Evaluation and Feedback subtotal**	**/100**	

9. **Epi InfoTM:** This software can be downloaded free of charge from the CDC website (http://www.cdc.gov/epiinfo/)

All reasonable precautions have been taken by the World Health Organization to verify the information contained in this document. However, the published material is being distributed without warranty of any kind, either expressed or implied. The responsibility for the interpretation and use of the material lies with the reader. In no event shall the World Health Organization be liable for damages arising from its use.
WHO acknowledges the Hôpitaux Universitaires de Genève (HUG), in particular the members of the Infection Control Programme, for their active participation in developing this material.

Hand Hygiene Self-Assessment Framework 2010

4. Reminders in the Workplace

Question	Answer	Score	WHO improvement tools
**4.1** Are the following posters (or locally produced equivalent with similar content) displayed?			→ Guide to Implementation II.4
4.1a Poster explaining the indications for hand hygiene **Choose one answer**	Not displayed	0	→ Your 5 Moments for Hand Hygiene (Poster)
	Displayed in some wards/treatment areas	15	
	Displayed in most wards/treatment areas	20	
	Displayed in all wards/treatment areas	25	
4.1b Poster explaining the correct use of handrub **Choose one answer**	Not displayed	0	→ How to Handrub (Poster)
	Displayed in some wards/treatment areas	5	
	Displayed in most wards/treatment areas	10	
	Displayed in all wards/treatment areas	15	
4.1c Poster explaining correct hand-washing technique **Choose one answer**	Not displayed	0	→ How to Handwash (Poster)
	Displayed in some wards/treatment areas	5	
	Displayed in most wards/treatment areas	7.5	
	Displayed at every sink in all wards/treatment areas	10	
**4.2** How frequently does a systematic audit of all posters for evidence of damage occur, with replacement as required? **Choose one answer**	Never	0	→ Guide to Implementation II.4
	At least annually	10	
	Every 2-3 months	15	
**4.3** Is hand hygiene promotion undertaken by displaying and regularly updating posters other than those mentioned above?	No	0	→ Guide to Implementation II.4
	Yes	10	
**4.4** Are hand hygiene information leaflets available on wards?	No	0	→ Hand Hygiene: When and How Leaflet → Guide to Implementation II.4
	Yes	10	
**4.5** Are other workplace reminders located throughout the facility? (e.g. hand hygiene campaign screensavers, badges, stickers, etc)	No	0	→ SAVE LIVES: Clean Your Hands Screensaver → Guide to Implementation II.4
	Yes	15	
	**Reminders in the Workplace subtotal**	**/100**	

All reasonable precautions have been taken by the World Health Organization to verify the information contained in this document. However, the published material is being distributed without warranty of any kind, either expressed or implied. The responsibility for the interpretation and use of the material lies with the reader. In no event shall the World Health Organization be liable for damages arising from its use.
WHO acknowledges the Hôpitaux Universitaires de Genève (HUG), in particular the members of the Infection Control Programme, for their active participation in developing this material.

5. Institutional Safety Climate for Hand Hygiene

Question	Answer	Score	WHO improvement tools
**5.1** With regard to a hand hygiene team[10] that is dedicated to the promotion and implementation of optimal hand hygiene practice in your facility:			→ Guide to Implementation II.5
5.1a Is such a team established?	No	0	
	Yes	5	
5.1b Does this team meet on a regular basis (at least monthly)?	No	0	
	Yes	5	
5.1c Does this team have dedicated time to conduct active hand hygiene promotion? (e.g. teaching monitoring hand hygiene performance, organizing new activities)	No	0	
	Yes	5	
**5.2** Have the following members of the facility leadership made a clear commitment to support hand hygiene improvement? (e.g. a written or verbal commitment to hand hygiene promotion received by the majority of health-care workers)			→ Template Letter to Advocate Hand Hygiene to Managers<br>→ Template Letter to communicate Hand Hygiene Initiatives to Managers<br>→ Guide to Implementation II.5
5.2a Chief executive officer	No	0	
	Yes	10	
5.2b Medical director	No	0	
	Yes	5	
5.2c Director of nursing	No	0	
	Yes	5	
**5.3** Has a clear plan for the promotion of hand hygiene throughout the entire facility for the 5 May (Save Lives Clean Your Hands Annual Initiative) been established ?	No	0	→ Sustaining Improvement – Additional Activities for Consideration by Health-Care Facilities<br>→ Guide to Implementation II.5
	Yes	10	
**5.4** Are systems for identification of Hand Hygiene Leaders from all disciplines in place?			
5.4a A system for designation of Hand Hygiene champions[11]	No	0	
	Yes	5	
5.4b A system for recognition and utilisation of Hand Hygiene role models[12]	No	0	
	Yes	5	
**5.5** Regarding patient involvement in hand hygiene promotion:			→ Guidance on Engaging Patients and Patient Organizations in Hand Hygiene Initiatives<br>→ Guide to Implementation II.5
5.5a Are patients informed about the importance of hand hygiene? (e.g. with a leaflet)	No	0	
	Yes	5	
5.5b Has a formalised programme of patient engagement been undertaken?	No	0	
	Yes	10	
**5.6** Are initiatives to support local continuous improvement being applied in your facility, for example:			→ Sustaining Improvement – Additional Activities for Consideration by Health-Care Facilities<br>→ Guide to Implementation II.5
5.6a Hand hygiene E-learning tools	No	0	
	Yes	5	
5.6b A hand hygiene institutional target to be achieved is established each year	No	0	
	Yes	5	
5.6c A system for intra-institutional sharing of reliable and tested local innovations	No	0	
	Yes	5	
5.6d Communications that regularly mention hand hygiene e.g. facility newsletter, clinical meetings	No	0	
	Yes	5	
5.6e System for personal accountability[13]	No	0	
	Yes	5	
5.6f A Buddy system[14] for new employees	No	0	
	Yes	5	
	**Institutional Safety Climate subtotal**	**/100**	

All reasonable precautions have been taken by the World Health Organization to verify the information contained in this document. However, the published material is being distributed without warranty of any kind, either expressed or implied. The responsibility for the interpretation and use of the material lies with the reader. In no event shall the World Health Organization be liable for damages arising from its use.
WHO acknowledges the Hôpitaux Universitaires de Genève (HUG), in particular the members of the Infection Control Programme, for their active participation in developing this material.

Hand Hygiene Self-Assessment Framework 2010

10. Hand hygiene team: The make-up of this team will vary. It is likely to most frequently consist of an infection control unit, but may range (depending on resources available) from a single person with the role of managing the hand hygiene programme, to a group of staff members from various departments within the facility with meetings dedicated to the hand hygiene programme.

11. Hand hygiene champion: A person who is an advocate for the causes of patient safety and hand hygiene standards and takes on responsibility for publicizing a project in his/her ward and/or facility-wide.

12. Hand hygiene role model: A person who serves as an example, whose behaviour is emulated by others. In particular, a hand hygiene role model should have a hand hygiene compliance rate of at least 80%, be able to remind others to comply, and be able to teach practically about the WHO 5 Moments for Hand Hygiene concept.

13. System for personal accountability: explicit actions are in place to stimulate health-care workers to be accountable for their behaviour with regard to hand hygiene practices. Examples are notification by observers or infection control professionals, reproaches by peers, and reports to higher level facility authorities, with possible consequences on the individual evaluation.

14. Buddy system: A programme in which each new health-care worker is coupled with an established, trained health-care worker who takes responsibility for introducing them to the hand hygiene culture of the health-care setting (including practical training on indications and technique for performing hand hygiene, and explanation of hand hygiene promotion initiatives within the facility).

All reasonable precautions have been taken by the World Health Organization to verify the information contained in this document. However, the published material is being distributed without warranty of any kind, either expressed or implied. The responsibility for the interpretation and use of the material lies with the reader. In no event shall the World Health Organization be liable for damages arising from its use.
WHO acknowledges the Hôpitaux Universitaires de Genève (HUG), in particular the members of the Infection Control Programme, for their active participation in developing this material.

Hand Hygiene Self-Assessment Framework 2010

Interpretation: A Four Step Process

1.
Add up your points.

Score	
**Component**	**Subtotal**
1. System Change	
2. Education and Training	
3. Evaluation and Feedback	
4. Reminders in the Workplace	
5. Institutional Safety Climate	
**Total**	

2.
Determine the assigned 'Hand Hygiene Level' for your facility.

Total Score (range)	Hand Hygiene Level
0 - 125	Inadequate
126 - 250	Basic
251 - 375	Intermediate (or Consolidation)
376 - 500	Advanced (or Embedding)

3.
If your facility has reached the Advanced level, then complete the Leadership section overleaf.

(otherwise go to Step 4).

4.
Review the areas identified by this evaluation as requiring improvement in your facility and develop an action plan to address them (starting with the relevant WHO improvement tools listed). Keep a copy of this assessment to compare with repeated uses in the future.

All reasonable precautions have been taken by the World Health Organization to verify the information contained in this document. However, the published material is being distributed without warranty of any kind, either expressed or implied. The responsibility for the interpretation and use of the material lies with the reader. In no event shall the World Health Organization be liable for damages arising from its use.
WHO acknowledges the Hôpitaux Universitaires de Genève (HUG), in particular the members of the Infection Control Programme, for their active participation in developing this material.

Hand Hygiene Self-Assessment Framework 2010

Leadership Criteria	Answer (circle one)	
**System Change**		
Has a cost-benefit analysis of infrastructure changes required for the performance of optimal hand hygiene at the point of care been performed?	Yes	No
Does alcohol-based handrubbing account for at least 80% of hand hygiene actions performed in your facility?	Yes	No
**Training and Education**		
Has the hand hygiene team undertaken training of representatives from other facilities in the area of hand hygiene promotion?	Yes	No
Have hand hygiene principles been incorporated into local medical and nursing educational curricula?	Yes	No
**Evaluation and Feedback**		
Are specific healthcare associated infections (HCAIs) monitored? (eg. *Staphylococcus aureus* bacteremia, Gram negative bacteremia, device-related infections)	Yes	No
Is a system in place for monitoring of HCAI in high risk-settings? (e.g. intensive care and neonatal units)	Yes	No
Is a facility-wide prevalence survey of HCAI performed (at least) annually?	Yes	No
Are HCAI rates presented to facility leadership and to health-care workers in conjunction with hand hygiene compliance rates?	Yes	No
Is structured evaluation undertaken to understand the obstacles to optimal hand hygiene compliance and the causes of HCAI at the local level, and results reported to the facility leadership?	Yes	No
**Reminders in the Workplace**		
Is a system in place for creation of new posters designed by local health-care workers?	Yes	No
Are posters created in your facility used in other facilities?	Yes	No
Have innovative types of hand hygiene reminders been developed and tested at the facility?	Yes	No
**Institutional Safety Climate**		
Has a local hand hygiene research agenda addressing issues identified by the WHO Guidelines as requiring further investigation been developed?	Yes	No
Has your facility participated actively in publications or conference presentations (oral or poster) in the area of hand hygiene?	Yes	No
Are patients invited to remind health-care workers to perform hand hygiene?	Yes	No
Are patients and visitors educated to correctly perform hand hygiene?	Yes	No
Does your facility contribute to and support the national hand hygiene campaign (if existing)?	Yes	No
Is impact evaluation of the hand hygiene campaign incorporated into forward planning of the infection control programme?	Yes	No
Does your facility set an annual target for improvement of hand hygiene compliance facility-wide?	Yes	No
If the facility has such a target, was it achieved last year?	Yes	No

**Total**	**/20**

Your facility has reached the Hand Hygiene Leadership level if you answered "yes" to at least one leadership criteria per category and its total leadership score is 12 or more. Congratulations and thank you!

All reasonable precautions have been taken by the World Health Organization to verify the information contained in this document. However, the published material is being distributed without warranty of any kind, either expressed or implied. The responsibility for the interpretation and use of the material lies with the reader. In no event shall the World Health Organization be liable for damages arising from its use.
WHO acknowledges the Hôpitaux Universitaires de Genève (HUG), in particular the members of the Infection Control Programme, for their active participation in developing this material.
